# Analysis of the relationship between structural pillars in the constitution of the psychism and the emergence of a place of enunciation in babies with and without delays in language acquisition

**DOI:** 10.1590/2317-1782/20212021296en

**Published:** 2022-10-17

**Authors:** Renata Souto Bolzan, Anaelena Bragança de Moraes, Ana Paula Ramos de Souza

**Affiliations:** 1 Departamento de Psicologia, Universidade Federal de Santa Maria – UFSM - Santa Maria (RS), Brasil.; 2 Departamento de Epidemiologia, Universidade Federal de Santa Maria – UFSM - Santa Maria (RS), Brasil.; 3 Departamento de Saúde da Comunicação Humana, Universidade Federal do Rio Grande do Sul – UFRGS - Porto Alegre (RS), Brasil

**Keywords:** Child Development, Language Development, Risk Factors, Mental Health, Psychoanalysis

## Abstract

**Purpose:**

To analyze the correlation between the psychism structuring pillar in the RIID (Reference Indicators of Infant Development) script and language acquisition through Enunciative Signs of Language Acquisition (ESLA) as to the role of the baby and the mother in early protoconversations.

**Methods:**

The sample included 77 infants, who completed all the RIID and ESLA assessments. The results obtained from these two instruments were analyzed using Spearman's coefficient and the Mann-Whitney U test, considering a p-value < 0.05.

**Results:**

A significant correlation was identified between changes in the indicators related to the maternal function (assumption of subject, establishment of demand and presence/absence alternation) and changes in the enunciative signals of babies and mothers. There was no significant correlation in the isolated relation of the paternal function pillar and the presence of delayed language acquisition.

**Conclusion:**

There was a significant correlation between the pillars of maternal function of the RIID script and language risk by ESLA.

## INTRODUCTION

In a perspective of identifying the existence of risk to the psychic constitution and the acquisition of language from the first years of a child's life, researchers have been studying and creating early detection protocols in the last years^(1,2)^. Some studies have identified a close relationship between risk to the psychic constitution and language acquisition in children^(3,4)^.

Based on psychoanalytic theory, a group of researchers developed Clinical Reference Indicators for Infant Development (CRIID), observable in the first 18 months of a child's life. This tool makes it possible to observe the mother-baby relationship and obtain a differentiated reading of the baby's psychic manifestations^(1)^. The set of 31 indicators of the CRDI (Chart 1) was built from the following theoretical pillars:

**Chart 1:** Clinical Indicators of Risk/Reference to Child Development (RIID)

Age Group	Indicators	Pillar
1 to 3 months and 29 days	1- When the child cries or screams, the mother knows what the child wants.	SA/DE
	2- The mother speaks to the child in a style particularly directed at her (motherese)	SA
	3- The child reacts to motherese	DE
	4- The mother proposes something to the child and waits for his reaction.	AP
	5- There are glances exchanged between the child and the mother.	SA/AP
4 to 7 months and 29 days	6- The child uses different signs to express his/her different needs.	DE
	7- The child reacts (smiles, vocalizes) when the mother or another person is addressing him/her.	DE
	8- The child actively seeks the mother's gaze.	DE/AP
8 to 11 months and 29 days	9-The mother perceives that some of the child's requests can be a way to get her attention.	SA/DE
	10- During body care, the infant actively seeks loving games and play with the mother.	DE
	11-- Mother and child share a particular language.	SA/AP
	12- The child strangers people he doesn't know.	PF
	13- The child makes jokes.	DE
	14- The child accepts semi-solid, solid and varied foods.	DE
12 to 18 months	15- The mother alternates moments of dedication to the child with other interests.	DE/PF
	16- The child tolerates the mother's brief absences well, and reacts to prolonged absences.	DE/PF
	17- The mother no longer feels obligated to satisfy everything the child asks.	PF
	18- The parents set small rules of behavior for the child.	PF

**Caption:** SA=subject assumption, DE= demand establishment, AP= alternating presence and absence, PF= Paternal Function

- Subject assumption (SA): which characterizes an anticipation made by the mother/or surrogate, of the presence of a psychic subject in the baby, not yet constituted. The baby's productions, even if involuntary, are taken as productions addressed to the mother. However, it is not enough that an adult assumes a subject, but also that he/she can recognize the manifestations of this child with its singularities. It is this assumption and this recognition by the mother that will make this baby, later on, come to fill the place of enunciation, as a speaker of language;
- Establishment of the demand (ED): refers to the baby's first involuntary reactions at birth, such as crying, which are recognized by the mother as a request that the child addresses to her. This demand will be the basis of all later language activity and relationships with others;
- Presence and absence alternation (AP): these are the maternal actions referring to the care she directs to the baby, not responding only with presence or absence, but producing an alternation. It is this alternation that will enable the baby to internalize and signify the difference me-not-me, supported by the mother's look, touch, and voice;
- Paternal function (PF): this function is what marks the maternal actions, occupying the third instance guided by the social dimension. This exercise of the paternal function on the mother-baby pair is what will make the symbolic separation between them, and allow the mother to disregard this baby as an object aimed only at her satisfaction^(1,2)^.

Regarding the language acquisition process^(2,5,6)^ the Enunciative Signs of Language Acquisition (ESLA) were developed, distributed in four phases, which evaluate the babies from the Benvenistean theoretical reference in language acquisition^(7)^. In this framework, three enunciative mechanisms are proposed that have a relation of logical anteriority. Therefore, it cannot be said that the first is suppressed or replaced by the second, and the second by the third mechanism, but that they can coexist^(8)^.

In the first mechanism the relations of conjunction and disjunction between mother and baby are observed, from the initial protoconversations. In it it is observed that the mother supports the baby in the protoconversation by requesting his participation in pre-established routines. Clapping hands to a song, vocalizing or babbling when summoned by the mother, are some examples of conjunction, and that, when experiencing the conjunction, the baby realizes that its manifestations have an effect on the other and, therefore, it initiates manifestations by gestures, vocalizations, looks that summon the other to the protoconversation or to the dialog (disjunction). These are dynamics in which the references (about what is being talked about) are in the gestuality, that is, they are shown^(8)^.

Subsequently, the passage from the shown to the spoken reference occurs, which refers to the second enunciative mechanism. The second enunciative mechanism is the semantization of language and the construction of reference by the dyad *I-you (I=child, you= usual allocuter*). Here emerge the nominations, the comments, the word combinations, the sense adjustments and the form of the relations produced in the enunciative relation constituted by I and you.

Finally, there is the installation of the subject in the discourse, which is the third enunciative mechanism, whose greatest occurrence is from the second year of life. In this enunciative mechanism, the child is established as a subject of discourse, and is already able to intimate, interrogate and imagine via language, as well as to mark itself in different forms of *I* instantiation (first name, use of the third person pronoun, use of the pronoun *I*)^(8)^.

Considering the amplitude and importance of the RIID theoretical pillars in the identification of psychic suffering and possible effects on language functioning, the present study aims to analyze the correlation of the CRIID (Clinical Risk Indicators/Child Development Reference) script theoretical pillars with the language acquisition process analyzed through Enunciative Signs of Language Acquisition (ESLA), regarding the role of the baby and the mother in the initial protoconversations.

## METHODS

This study is part of a research project called "Comparative analysis of the development of preterm and full-term babies with and without psychological risk: from detection to intervention", approved in May 2014 by the Comitê de Ética em Pesquisa of the Universidade Federal de Santa Maria under CAAE number 28586914.0.00005346.

The research had an initial sample of 182 babies, born at term and preterm, healthy, without injuries or suspected syndromes. Mothers and their infants were contacted and invited to participate in the research through a Unidade Básica de Saúde (UBS), when they arrived for the heel prick. After reading and signing the Termo de Consetimento Livre e Esclarecido (TCLE), an initial interview was conducted, which involved a collection of obstetric, sociodemographic, and psychosocial data. During the remaining evaluations, continued interviews were conducted to observe changes or not in psychosocial and sociodemographic aspects.

These babies were evaluated by a multidisciplinary team, composed of a pediatrician, physiotherapist, speech therapist, psychologist, and occupational therapist. Of these 182 babies who entered the research, only 77 babies completed all the evaluations at 24 months, and became the sample for this research. The reduction in the sample is due to the fact that the babies did not return for the evaluations, especially after the second semester of life. According to some mothers, this dropout occurred when they assessed that their children were developing well, or even when they returned to work, making it difficult to keep going to the health unit for the continuity of the evaluation.

The age ranges for collection were six, corresponding to the meetings in which the evaluations were carried out with the babies, following the application ranges of the instruments, as foreseen in their design: range 1-3 months and 1 day to 4 months and 29 days; range 2-5 months and 1 day to 6 months and 29 days; range 3-7 months and 1 day to 9 months and 29 days; range 4-11 months and 1 day to 12 months and 29 days, range 5- 17 months and 1 day to 18 months and 29 days, and range 6- 23 months and 1 day to 24 months and 29 days.

In these collections, the infants were filmed for 15 minutes in interaction with their mothers, in which they were instructed to sing (3 minutes), talk (3 minutes), and play with toy (3 minutes). During the first 9 minutes, the babies were seated in a baby comfort chair, and then for the last 6 minutes they were free to interact, lying prone and supine, in the first two age groups. In age group 3, the baby could already be on the tatami and the procedure for filming was the same. In age groups 4, 5 and 6, they were offered a box of toys that were appropriate for the child's age and easy to clean, so that they could freely explore sitting or moving on the tatami for 15 minutes. In age groups 5 and 6, the examiner came in during the last 5 minutes to analyze the child's reaction to his or her presence from the enunciative point of view.

### Instruments and analysis

Two instruments were applied in the analysis of the footage, the Infant Development Risk/Reference Indicators (RIID) script, short version^(2)^ and the Enunciative Signs of Language Acquisition (ESLA)^(2,5-7)^. Infants are considered at risk when two or more indicators are missing from the RIID script^(2)^. Below is Table 1, with the RIID script, reduced version, used in the analysis of this study.

**Table 1:** Spearman's correlation coefficient and statistical significance

Variables	Correlation coefficient (r)	p-value*
ESLA baby x ESLA mother	0.76	0.000
ESLA baby x RIID- SA	0.31	0.006
ESLA baby x RIID- DE	0.29	0.011
ESLA baby x RIID-AP	0.23	0.048
ESLA baby x RIID-PF	0.09	0.460
ESLA baby x TOTAL RIID	0.23	0.044
ESLA mother x RIID-SA	0.36	0.001
ESLA mother x RIID-ED	0.34	0.003
ESLA mother x RIID-AP	0.31	0.006
ESLA mother x RIID-PF	0.18	0.118
ESLA mother x TOTAL RIID	0.31	0.006
TOTAL ESLA x RIID-SA	0.34	0.002
TOTAL ESLA x RIID-DE	0.31	0.006
TOTAL ESLA x RIID-AP	0.26	0.020
TOTAL ESLA x RIID-PF	0.11	0.338
TOTAL ESLA x TOTAL RIID	0.27	0.020

* Spearman's correlation coefficient

**Caption:** ESLA=enunciative signs of language acquisition; RIID=clinical indicators of child development referral, SA=subject assumption; DE=demand establishment, AP=presence/absence, PF=paternal function. (n=77) p≤0.05

The analysis of the RIID script indicators was performed during the continued interview and in the observation of the videos, and then checked by the research supervisor also through the videos. Below is Chart 2, with the description of the ESLA.

**Chart 2:** Enunciative Signs of Language Acquisition (ESLA)

**Phase I - Signs from 2 to 6 months and 29 days**	Category
1. Child reacts to *motherese*, through vocalizations, body movements, or gaze.	Baby
2. The infant fills his place in the interlocution with verbal sounds such as vowels and/or consonants. (for example, /a, u, i/ or /m n p t/).	Baby
**3. The child fills his/her place in the interlocution with non-verbal sounds in a manner attuned to the enunciative context (smiling, screaming, crying, coughing, grunting).**	Baby
**4. The child fills his place in the interlocution silently only with body movements and looks attuned to the enunciative context.**	Baby
5. The child initiates the conversation or protoconversation	Baby
6. Child and mother (or surrogate) exchange glances during interaction (for 3 or more seconds)	Baby and Mother
**7. Mother (or surrogate) assigns meaning to infant's verbal and nonverbal manifestations, sustaining the protoconversation.**	Mother
8. Mother (or surrogate) uses motherese by talking to the infant in a manner attuned to what is happening in the context and waiting for the infant's responses.	Mother
**Phase II - Signs from 7 to 12 months and 29 days**	
9. The infant fills her place in the interlocution (enunciation) with verbal sounds (syllables with varied vowels and consonants-at least two points and two consonant articulation modes-for example, syllables like pa, ta, ma, na etc.) and in a manner addressed to the interlocutor	Baby
10. The child outlines the production of protowords by mirroring the mother's (or surrogate's) speech, addressing her production to the interlocutor.	Baby
**11. The child drafts the production of protowords spontaneously, addressing them to the interlocutor.**	Baby
**12. The mother responds to the child, pauses and gives space for new manifestation by the child.**	Mother
**Phase III - Signs 13 to 17 months**	
13. The child names spontaneously and intelligibly to the adult interlocutor, objects that are absent in the context.	Baby
**14. The child produces an utterance not understood by the adult, but makes an effort to make himself understood by altering prosody, intonation, rhythm or repetition to try to be understood.**	Baby
**15. The child names spontaneously and intelligibly to the adult interlocutor, objects, people, actions, which are present in the enunciative context.**	Baby
16. The child makes gestures to try to make herself understood when the adult interlocutor does not understand her	Baby
**17. The child repeats the interlocutor adult's utterance as a way of organizing or reorganizing his utterance, for example, by improving the syntactic form, or phonological, or lexical item choice, or even by accentuating some item prosodically.**	Baby
**18. The child talks to different adult interlocutors (father, mother, examiner).**	Baby
**19. The adult interlocutor assigns possible meaning to the child's verbal productions, i.e., in a attuned manner.**	Mother
**Phase IV- Signs 18 to 24 months**	
**20. The child requests objects and/or asks for clarification from the interlocutor adult, marking his position as a speaker.**	Baby
**21.** T**he child uses distinct phonemic forms to convey different meanings in his utterance (at least two articulatory points-labial (b, p, m) and alveolar (t, d, n) -and two distinct consonantal sound classes-at least nasal (m, n) and plosive (p, t).**	Baby
**22. The child uses distinct forms (words) to convey different meanings in his utterance.**	Baby
**23. Child combines words, in direct or inverse form, to convey different meanings (short sentences or compound expressions)**	Baby
**24. When the child presents verbal productions different from adult speech, the adult interlocutor reacts by making a neutral repair request (what) or by correctly repeating the child's speech, without breaking the dialogue.**	Mother

Source: In **bold** are the items that showed the strongest statistically in factor analysis in separating groups of infants (Fattore et al.^(2)^, Crestani et al.^(5)^, Crestani et al.^(6)^)

The ESLA was analyzed by viewing the videos, by three speech therapists specialized in language acquisition, and checked by the research supervisor. There was greater than 95% agreement in the assignment of the signs^(2,5,6)^. To build the analysis of this research, we also considered the distinctions as to the enunciative signs related to the baby (the way it occupies its place of enunciation) and to the mother (the way it supports the child's place of enunciation), to consider the variables intended in the correlation and comparison between the results of the RIID script and ESLA.

The results obtained with the instruments were entered into an Excel spreadsheet and then the analyses foreseen in the research objectives were carried out: of correlation between RIID theoretical pillars and ESLA results, distinguishing the signs related to the way the baby occupies its place of enunciation and the signs related to the enunciative support offered by the mother.

For the statistical analysis, Spearman's correlation coefficient was used (Table 1), to verify the correlations between the enunciative signs and the RIID indicators, considering the following variables:

- ESLA of the baby x ESLA of the mother;
- Baby's ESLA x indicators of the Subject Assumption pillar;
- ESLA of the Baby x indicators of the Demand Establishment pillar;
- ESLA of Baby x indicators of the Alternation between Presence and Absence axis;
- Baby's ESLA x indicators of the Paternal Function pillar;
- Baby's ESLA x RIID Total;
- Mother's ESLA x indicators from the Subject Assumption pillar;
- Mother's ESLA x indicators from the Demand Establishment pillar;
- Mother's ESLA x indicators from the Alternation between Presence and Absence pillar;
- Mother's ESLA x indicators from the Paternal Function pillar;
- Mother's ESLA x Total RIID;
- Total ESLA x indicators from the Subject Assumption pillar;
- ESLA total x indicators from the Demand Establishment pillar;
- ESLA total x indicators from the Alternation between Presence and Absence pillar;
- total ESLA x indicators from the Paternal Function pillar; and
- total ESLA x total RIID.

In addition to this analysis, data from infants with (less than 18 signs) and without language acquisition delay (18 or more signs)^(7)^ were compared as to their performance on the pillars of the RIID script (SA, ED, PA, PF) and the total RIID by means of the Mann-Whitney U-test. It is worth noting that it was from an initial clinical study that it was observed that children with 18 or more enunciative signs had no language acquisition delay at 24 months. However, children with delayed language acquisition had 12 enunciative signs out of the 24 signs evaluated^(7)^

## RESULTS

After analyzing the data from the 77 infants, it was observed that there was a higher number of infants who presented risk by the ESLA (45 infants), when compared to the RIID script (31 infants). Table 1 shows the correlation analysis between both instruments.

It can be seen in Table 1 that there is a significant correlation between the ESLA of the infant and the ESLA of the mother, confirming the relationship between the maternal and the infant factor in generating delayed language acquisition. This observation is confirmed in the correlation between the total ESLA, the baby's total ESLA, and the mother's total ESLA.

In relation to the pillars of the RIID script and the ESLA, it is observed that the pillars subject assumption (SS), demand establishment (DE), and alternation between presence and absence (PA) correlated with both maternal and infant signals. There is no significant correlation between the enunciative signals and the paternal function pillar in isolation. There was also a significant correlation between Total ESLA and Total RIID.

Therefore, the correlation was confirmed between the indicators related to the pillars of subject assumption, establishment of demand, and alternation between presence and absence, which are fundamentally related to the maternal function.

Table 2 presents the comparison of the RIID pillar scores between the ESLA groups.

**Table 2:** Comparison of RIID Pillar Scores among ESLA Groups

	Averages		
	ESLA <18	ESLA ≥18	p-value*
RIID SA	4.24	4.66	0.021
RIID DE	9.29	10.19	0.034
RIID AP	3.11	3.56	0.053
RIID PF	3.84	4.16	0.631
TOTAL RIID	14.87	16.31	0.084

* Mann-Whitney U-test; p≤0.05

**Caption:** RIID=referenced clinical indicators of child development, ESLA=enunciative signs of language acquisition. ESLA cutoff point= 18 or more normal, <18 = risk, SA=subject assumption, DE=demand establishment, AP=presence/absence, PF=Paternal Function

Considering that the cutoff point for delayed language acquisition in ESLA is 18 signs^(7)^, it is observed in Table 2 that there was a significant difference in the scores of the pillars of the RIID script between the groups with and without delayed language acquisition as to the pillars of subject assumption (SA), demand establishment (DE) and presence/absence alternation (PA) of the clinical indicators of reference to child development.

In addition, it was possible to identify that there is no significant difference in the scores of the paternal function (PF) pillar for cases in which the infant presents fewer or more enunciative signs of language acquisition, as well as the total value of the RIID in this analysis, which, in a way, is in line with what was identified in the analysis presented in Table 1. Thus, it can be stated that the maternal function presented itself especially related to the occupation of a place of enunciation by the babies and/or sustaining of this place by their mothers.

## DISCUSSION

The results of this research, involving Baby's ESLA, Mother's ESLA versus total ESLA, showed a relation of the mother and baby's signals in the generation of delayed language acquisition, i.e., the hypothesis that the generation of delayed language acquisition is due only to the baby's biological conditions is no longer valid. The enunciative support of the baby is as important as the biological limitations in the generation of language acquisition delay. In particular, it is emphasized that the nurturing of the speaking assumption by the caregiver^(9)^ is critical for the infant to engage in early conjunctive relations and to discover the effects of its manifestations on the other through disjunctive relations^(8)^.

In the correlation and comparison between cases with delay and without delay in language acquisition identified by ESLA, changes in the three pillars of maternal function SA, DE, PA of the RIID script were observed in both the infant's and mother's signs. This indicates that the assumption of a subject, the establishment of the baby's demand, and the alternation between presence and absence are key in creating a place of enunciation for the baby^(7)^. The results indicate that when assuming a subject, the mother also manages to assume a speaker^(9)^, making room for interpreting the baby's gestures and vocalizations^(10)^, in addition to her demands and keeping room for the next manifestations of this subject, evidenced in the alternation between absence and presence^(11)^.

As for indicator 4 of the RIID script (the mother proposes something and waits for the baby's response), it can be said that it is fundamental in the generation of dialog^(12)^, which highlights the importance of the mother or her substitute not being only presence or absence. This pillar seems to be necessary for the baby to emerge in the disjunction relation, because the mother needs to leave vacant a discursive place for the baby and he has to occupy this place to emerge in speech.

On the other hand, the paternal function pillar did not present itself as an isolated generator of the delay in language acquisition, but operated in the other two pillars, because the exercise of the maternal function is always referenced to the paternal function^(13)^. This means that when isolating the pillars of the RIID corresponding to the maternal function (castrated mother), the paternal function also operates, although its discrimination from the maternal function is more evident in the second year of life.

One study^(14)^ observed that infants with alterations in the paternal function pillar showed greater difficulty in having speech detached from the maternal speech, but not a difficulty in speaking itself, that is, in moving from the shown to the spoken reference. The greatest difficulty was in reaching the third enunciative mechanism, or the entry into one's own speech, which is not evaluated in the ESLA, since it is an activity somewhat more present from 24 months on in babies, when language is used to imagine a possible world and the instantiations of oneself in speech are observed, as well as, the apparatus of functions^(8)^. Perhaps, at an age above two years it would be possible to observe other effects of the paternal function on discursive autonomy, which were not possible to analyze in this study.

Thus, the relationship between psychism and language demands that a hypothesis of language functioning be raised, which includes both psychic aspects and linguistic aspects. The way parents support the baby during the dialog is affected by the way they suppose the child. If the child is not assumed as a separate subject, this leads to a language functioning in which the mother can speak for the child, without giving the child a place of enunciation. On the other hand, if the son has difficulties in occupying his place of enunciation, the parents may speak for him, due to anxiety in the face of the child's quieter position^(14)^.

Finally, it is worth mentioning the limitations of this study due to the use of the reduced RIID script, although it had a good longitudinal sample. Thus, the use of a larger sample is recommended for a more representative analysis of the population. It is noteworthy that this general statistical trend does not exclude the possibility of singular cases, in which only the altered paternal function may generate a more significant delay in language acquisition, already detectable in the second year of life.

It is also emphasized the importance of a follow-up by both a speech therapist and a psychoanalyst from the first year of the child's life, because in this way it is possible to recognize warning signs that can be observed since the first months of life, by the interaction between the baby and the parental figures. In addition, for the performance of early detection of risk to child development, an interdisciplinary contribution is necessary, in a perspective of health promotion, which aims at the subject's quality of life, since its birth^(15)^.

## CONCLUSION

This work sought to analyze the correlation of the theoretical pillars of the Reference Indicators of Infant Development (RIID) and the possible differences in the Enunciative Signs of Language Acquisition (ESLA), regarding the role of the infant and the mother in the initial protoconversations. From the developed analysis, it can be stated that there was significant correlation between the pillars of subject assumption, demand establishment, and alternation between presence and absence of the IRDI script and language risk by ESLA. When compared, infants with and without delay in language acquisition by ESLA, the same pillars remained significant in differentiating the infants.
