# Supplementary material for: Analysis of the relationship between structural pillars in the constitution of the psychism and the emergence of a place of enunciation in babies with and without delays in language acquisition
Source: Codas. 2022 Oct 17;35(1):e20210296. doi: 10.1590/2317-1782/20212021296en (PMC10010426; doi:10.1590/2317-1782/20212021296en)
Supplement: Translation

# Translation: Análise da relação entre eixos estruturantes na constituição do psiquismo e emergência de um lugar de enunciação de bebês com e sem atraso na aquisição da linguagem

**DOI:** 10.1590/2317-1782/20212021296pt

**Authors:** Renata Souto Bolzan, Anaelena Bragança de Moraes, Ana Paula Ramos de Souza

**Affiliations:** 1 Departamento de Psicologia, Universidade Federal de Santa Maria – UFSM - Santa Maria (RS), Brasil.; 2 Departamento de Epidemiologia, Universidade Federal de Santa Maria – UFSM - Santa Maria (RS), Brasil.; 3 Departamento de Saúde da Comunicação Humana, Universidade Federal do Rio Grande do Sul – UFRGS - Porto Alegre (RS), Brasil.

**Keywords:** Desenvolvimento Infantil, Desenvolvimento da Linguagem, Fatores de Risco, Saúde Mental, Psicanálise

## Abstract

**Objetivo:**

Analisar a correlação entre os eixos estruturantes do psiquismo no roteiro IRDI (Indicadores de Referência ao Desenvolvimento Infantil) e na aquisição da linguagem por meio dos Sinais Enunciativos de Aquisição da Linguagem (SEAL) quanto ao papel do bebê e da mãe nas protoconversações iniciais.

**Método:**

A amostra contou com 77 bebês, que concluíram todas as avaliações no IRDI e no SEAL. Os resultados obtidos por meio desses dois instrumentos foram analisados por meio do coeficiente de Spearman e do teste de U de Mann-Whitney, considerando valor de p<= 0,05.

**Resultados:**

Identificou-se correlação significativa entre as alterações nos indicadores relacionados à função materna (suposição de sujeito, estabelecimento da demanda e alternância presença/ausência) e as alterações nos sinais enunciativos dos bebês e das mães. Não houve correlação significativa na relação isolada do eixo função paterna e a presença de atraso na aquisição da linguagem.

**Conclusão:**

Houve correlação significativa entre os eixos de função materna do roteiro IRDI e risco à linguagem pelo SEAL.

## INTRODUÇÃO

Em uma perspectiva de identificar a existência de risco à constituição psíquica e à aquisição da linguagem, desde os primeiros anos de vida de uma criança, pesquisadores têm-se debruçado a estudar e criar protocolos de detecção precoce nos últimos anos^(1,2)^. Alguns estudos identificaram uma relação próxima entre o risco à constituição psíquica e à aquisição da linguagem em crianças^(3,4)^.

Com base na teoria psicanalítica, um grupo de pesquisadores desenvolveu Indicadores Clínicos de Referência para o Desenvolvimento Infantil (IRDI), observáveis nos primeiros 18 meses de vida da criança. Esse instrumento possibilita observar a relação mãe-bebê e obter uma leitura diferenciada das manifestações psíquicas do bebê^(1)^. O conjunto de 31 indicadores do IRDI (Quadro 1) foi construído a partir dos seguintes eixos teóricos:

**Quadro 1:** Indicadores Clínicos de Risco/Referência ao Desenvolvimento Infantil (IRDI)

Faixa Etária	Indicadores	Eixos
1 a 3 meses e 29 dias	1- Quando a criança chora ou grita, a mãe sabe o que ela quer.	SS/ED
	2- A mãe fala com a criança num estilo particularmente dirigido a ela (*manhês*).	SS
	3- A criança reage ao *manhês*.	ED
	4- A mãe propõe algo à criança e aguarda a sua reação.	PA
	5- Há trocas de olhares entre a criança e a mãe.	SS/PA
4 a 7 meses e 29 dias	6- A criança utiliza sinais diferentes para expressar suas diferentes necessidades.	ED
	7- A criança reage (sorri, vocaliza) quando a mãe ou outra pessoa está se dirigindo a ela.	ED
	8- A criança procura ativamente o olhar da mãe.	ED/PA
8 a 11 meses e 29 dias	9- A mãe percebe que alguns pedidos da criança podem ser uma forma de chamar a sua atenção.	SS/ED
	10- Durante os cuidados corporais, a criança busca ativamente jogos e brincadeiras amorosas com a mãe.	ED
	11- Mãe e criança compartilham uma linguagem particular.	SS/PA
	12- A criança estranha pessoas desconhecidas para ela.	FP
	13- A criança faz gracinhas.	ED
	14- A criança aceita alimentação semissólida, sólida e variada.	ED
12 a 18 meses	15- A mãe alterna momentos de dedicação à criança com outros interesses.	ED/FP
	16- A criança suporta bem as breves ausências da mãe e reage às ausências prolongadas.	ED/FP
	17- A mãe já não se sente mais obrigada a satisfazer tudo que a criança pede.	FP
	18- Os pais colocam pequenas regras de comportamento para a criança.	FP

Fonte: SS=suposição de sujeito, ED= estabelecimento da demanda, PA= alternância de presença e ausência, FP= Função Paterna

- Suposição de sujeito (SS): que caracteriza uma antecipação realizada pela mãe/ou substituto, da presença de um sujeito psíquico no bebê, ainda não constituído. As produções do bebê, ainda que involuntárias, são tomadas como produções endereçadas à mãe. Porém, não basta apenas que um adulto suponha um sujeito, mas também que ele possa reconhecer as manifestações dessa criança com suas singularidades. Será essa suposição e esse reconhecimento da mãe que fará com que esse bebê, posteriormente, venha preencher o lugar de enunciação, como falante da língua;
- Estabelecimento da demanda (ED): refere-se às primeiras reações involuntárias do bebê ao nascer, como o choro, e que são reconhecidas pela mãe como um pedido que a criança lhe dirige. Essa demanda estará na base de toda atividade posterior de linguagem e de relação com os outros;
- Alternância de presença e ausência (PA): são as ações maternas referentes aos cuidados que dirige ao bebê, não respondendo apenas com presença ou ausência, mas produzindo uma alternância. Será essa alternância que possibilitará ao bebê a internalização e a significação da diferença eu-não eu, amparado pelo olhar, toque e voz materna;
- Função paterna (FP): essa função é o que baliza as ações maternas, ocupando a terceira instância orientada pela dimensão social. Esse exercício da função paterna sobre o par mãe-bebê é o que fará a separação simbólica entre eles, e permitirá a mãe desconsiderar esse bebê como um objeto voltado unicamente para sua satisfação^(1,2)^.

Em relação ao processo de aquisição da linguagem^(2,5,6)^ foram desenvolvidos os Sinais Enunciativos de Aquisição da Linguagem (SEAL), distribuídos em quatro fases, as quais avaliam os bebês a partir do referencial teórico benvenisteano em aquisição da linguagem^(7)^. Nesse referencial, três mecanismos enunciativos são propostos e possuem uma relação entre si de anterioridade lógica. Por isso, não se pode dizer que o primeiro é suprimido ou substituído pelo segundo, e o segundo pelo terceiro mecanismo, mas que eles podem coexistir^(8)^.

No primeiro mecanismo são observadas as relações de conjunção e disjunção entre mãe e bebê, desde as protoconversações iniciais. Nele é observado que a mãe sustenta o bebê na protoconversação, solicitando sua participação em rotinas pré-estabelecidas. Bater palmas para uma música, vocalizar ou balbuciar por convocação da mãe, são alguns exemplos de conjunção, e que, ao vivenciar a conjunção o bebê percebe que suas manifestações têm efeito no outro e, por isso, ele inicia manifestações por gestos, vocalizações, olhares que convocam o outro à protoconversação ou ao diálogo (disjunção). São dinâmicas em que as referências (sobre o que se fala) estão na gestualidade, ou seja, são mostradas^(8)^.

Posteriormente, ocorre a passagem da referência mostrada à falada, que se refere ao segundo mecanismo enunciativo. O segundo mecanismo enunciativo é a semantização da língua e a construção da referência pela díade *eu-tu* (*eu=criança, tu= alocutário usual*). Emergem, aqui, as nomeações, os comentários, as combinações de palavras, os ajustes de sentido e a forma das relações produzidas na relação enunciativa constituída por *eu* e por *tu.*

Por fim, há a instalação do sujeito no discurso, que é o terceiro mecanismo enunciativo, cuja ocorrência maior é a partir do segundo ano de vida. Nesse mecanismo enunciativo, a criança instaura-se como sujeito do discurso, e já é capaz de intimar, interrogar e imaginar via linguagem, bem como, se marcar em diferentes formas de instanciação EU (nome próprio, uso do pronome terceira pessoa, uso do pronome EU)^(8)^.

Considerando a amplitude e importância dos eixos teóricos do IRDI na identificação do sofrimento psíquico e possíveis efeitos no funcionamento de linguagem, o presente estudo objetiva analisar a correlação dos eixos teóricos do roteiro IRDI (Indicadores Clínicos de Risco/Referência ao Desenvolvimento Infantil) com o processo de aquisição da linguagem analisado por meio dos Sinais Enunciativos de Aquisição da Linguagem (SEAL), quanto ao papel do bebê e da mãe nas protoconversações iniciais.

## MÉTODO

Este estudo insere-se em um projeto de pesquisa denominado de “Análise comparativa do desenvolvimento de bebês prematuros e a termo, com e sem risco psíquico: da detecção à intervenção”, aprovado no mês de maio, do ano de 2014, pelo Comitê de Ética em Pesquisa, da Universidade Federal de Santa Maria sob o número de CAAE: 28586914.0.00005346.

A pesquisa contou com uma amostra inicial de 182 bebês, nascidos a termo e pré-termo, saudáveis, sem lesões ou suspeitas de síndromes. As mães e seus bebês foram contatados e convidados a participar da pesquisa por meio de uma Unidade Básica de Saúde (UBS), quando chegaram para realizar o teste do pezinho. Após a leitura e a assinatura do Termo de Consentimento Livre e Esclarecido (TCLE), foi realizada uma entrevista inicial, que envolveu uma coleta de dados obstétricos, sociodemográficos e psicossociais. Durante as demais avaliações foram realizadas entrevistas continuadas, para observação de mudanças ou não nos aspectos psicossociais e sociodemográficos.

Esses bebês foram avaliados por uma equipe multidisciplinar, composta por pediatra, fisioterapeuta, fonoaudiólogo, psicólogo e terapeuta ocupacional. Desses 182 bebês que ingressaram na pesquisa, apenas 77 bebês concluíram todas as avaliações aos 24 meses, e passaram a constituir a amostra desta pesquisa. A diminuição da amostra deve-se ao não retorno para as avaliações, sobretudo a partir do segundo semestre de vida. Conforme relato de algumas mães, esta desistência ocorreu quando elas avaliaram que seus filhos estavam com um bom desenvolvimento, ou ainda quando retornaram ao trabalho, sendo difícil manter a ida à unidade de saúde para a continuidade da avaliação.

As faixas etárias de coleta foram seis, correspondentes aos encontros em que foram realizadas as avaliações com os bebês, seguindo as faixas de aplicação dos instrumentos, conforme previsto em sua concepção: faixa 1- 3 meses e 1 dia a 4 meses e 29 dias; faixa 2- 5 meses e 1 dia a 6 meses e 29 dias; faixa 3- 7 meses e 1 dia a 9 meses e 29 dias; faixa 4- 11 meses e 1 dia a 12 meses e 29 dias, faixa 5- 17 meses e 1 dia a 18 meses e 29 dias e faixa 6- 23 meses e 1 dia a 24 meses e 29 dias.

Nessas coletas, os bebês foram filmados por 15 minutos em interação com suas mães, em que elas eram instruídas a cantar (3 minutos), conversar (3 minutos) e brincar com brinquedo (3 minutos). Durante os primeiros 9 minutos, os bebês ficavam sentados em uma cadeira bebê conforto, e, posteriormente, durante os 6 últimos minutos ficavam livres para a interação, deitados em prono e supino, nas duas primeiras faixas etárias. Na faixa etária 3, o bebê já poderia estar no tatame e o procedimento para filmagem era o mesmo. Nas faixas etárias 4, 5 e 6 era oferecida uma caixa de brinquedos adequados à idade da criança e de fácil higienização, para que explorassem livremente sentados ou se movimentando no tatame, durante 15 minutos. Nas faixas etárias 5 e 6, o examinador entrava nos últimos 5 minutos para analisar a reação da criança em relação à sua presença do ponto de vista enunciativo.

### Instrumentos e análises

Dois instrumentos foram aplicados na análise das filmagens, o roteiro de Indicadores de Risco/Referência ao Desenvolvimento Infantil (IRDI), versão reduzida^(2)^ e os Sinais Enunciativos de Aquisição da Linguagem (SEAL)^(2,5-7)^. Considera-se que os bebês estejam em risco quando dois ou mais indicadores estiverem ausentes no roteiro IRDI^(2)^. A seguir, consta o Quadro 1, com o roteiro IRDI, versão reduzida, utilizada na análise deste estudo.

A análise dos indicadores do roteiro IRDI foi realizada durante a entrevista continuada e na observação dos vídeos, e depois conferida pela orientadora da pesquisa também por meio dos vídeos. A seguir, consta o Quadro 2, com a descrição do SEAL.

**Quadro 2:** Sinais Enunciativos de Aquisição da Linguagem (SEAL)

**Fase I - Sinais de 2 a 6 meses e 29 dias**	Categoria
1. A criança reage ao *manhês*, por meio de vocalizações, movimentos corporais ou olhar.	Bebê
2. A criança preenche seu lugar na interlocução com sons verbais como vogais e/ou consoantes. (por exemplo, / a, u,i/ ou / m n p t/)	Bebê
**3. A criança preenche seu lugar na interlocução com sons não verbais de modo sintonizado ao contexto enunciativo (sorriso, grito, choro, tosse, resmungo).**	Bebê
**4. A criança preenche seu lugar na interlocução silenciosamente apenas com movimentos corporais e olhares sintonizados ao contexto enunciativo.**	Bebê
5. A criança inicia a conversação ou protoconversação.	Bebê
6. A criança e a mãe (ou sua substituta) trocam olhares durante a interação (por 3 ou mais segundos)	Bebê e Mãe
**7. A mãe (ou substituta) atribui sentido às manifestações verbais e não verbais do bebê, sustentando a protoconversação.**	Mãe
8. A mãe (ou substituta) utiliza o *manhês* falando com a criança de modo sintonizado ao que está acontecendo no contexto e aguardando as respostas do bebê.	Mãe
**Fase II - Sinais de 7 a 12 meses e 29 dias**	
9. A criança preenche seu lugar na interlocução (enunciado) com sons verbais (sílabas com vogais e consoantes variadas – ao menos dois pontos e dois modos articulatórios de consoantes- por exemplo, sílabas como pa, ta, ma, na etc) e de modo endereçado ao interlocutor	Bebê
10. A criança esboça a produção de protopalavras por espelhamento à fala da mãe (ou substituto), endereçando sua produção ao interlocutor.	Bebê
**11. A criança esboça a produção de protopalavras espontaneamente, endereçando-as ao interlocutor.**	Bebê
**12. A mãe responde à criança, faz pausa e dá espaço para nova manifestação da criança.**	Mãe
**Fase III - Sinais 13 a 17 meses**	
13. A criança nomeia de modo espontâneo e inteligível ao adulto interlocutor, objetos que estão ausentes no contexto.	Bebê
**14. A criança produz um enunciado não compreendido pelo adulto, mas se esforça por se fazer entender alterando prosódia, entonação, ritmo ou repetindo para tentar ser compreendida.**	Bebê
**15. A criança nomeia de modo espontâneo e inteligível ao adulto interlocutor, objetos, pessoas, ações, que estão presentes no contexto enunciativo.**	Bebê
16. A criança faz gestos para tentar fazer-se entender quando o adulto interlocutor não a compreende.	Bebê
**17. A criança repete o dizer do adulto interlocutor como forma de organizar ou reorganizar sua enunciação, por exemplo, aprimorando a forma sintática, ou fonológica, ou a escolha do item lexical ou mesmo acentuando algum item prosodicamente.**	Bebê
**18. A criança conversa com diferentes interlocutores adultos (pai, mãe, examinador).**	Bebê
**19. O adulto interlocutor atribui um sentido possível às produções verbais da criança, ou seja, de modo sintonizado.**	Mãe
**Fase IV- Sinais 18 a 24 meses**	
**20. A criança solicita objetos e/ou pede esclarecimentos ao adulto interlocutor, marcando sua posição como locutor.**	Bebê
**21. A criança utiliza formas fonêmicas distintas para veicular sentidos diferentes em sua enunciação (ao menos dois pontos articulatórios – labial (b,p,m) e alveolar (t,d,n)- e duas classes sonoras consonantais distintas – ao menos nasais (m,n) e plosivas (p,t).**	Bebê
**22. A criança utiliza distintas formas (palavras) para veicular sentidos diferentes em sua enunciação.**	Bebê
**23. A criança combina palavras, na forma direta ou inversa, para veicular sentidos diferentes (pequenas frases ou expressões compostas)**	Bebê
**24. Quando a criança apresenta produções verbais distintas da fala adulta, o adulto interlocutor reage fazendo um pedido de reparo neutro (o que) ou repetindo corretamente a fala infantil, sem romper o diálogo.**	Mãe

Fonte: Em **negrito** estão os itens que se apresentaram mais fortes estatisticamente na análise fatorial na separação de grupos de bebês (Fattore et al.^(2)^, Crestani et al.^(5)^, Crestani et al.^(6)^).

O SEAL foi analisado por meio da visualização dos vídeos, por três fonoaudiólogas especialistas em aquisição da linguagem, e conferido pela orientadora do trabalho. Houve concordância superior a 95% na atribuição dos sinais^(2,5,6)^. Para a construção da análise desta pesquisa, também foram consideradas as distinções quanto aos sinais enunciativos que se relacionarem ao bebê (modo como ocupa seu lugar de enunciação) e à mãe (modo como sustenta o lugar de enunciação do filho), para considerar as variáveis intencionadas na correlação e na comparação entre os resultados do roteiro IRDI e do SEAL.

Os resultados obtidos com os instrumentos foram lançados em uma planilha de *Excel* e, então, foram realizadas as análises previstas nos objetivos da pesquisa: de correlação entre eixos teóricos do IRDI e resultados do SEAL, distinguindo os sinais relacionados ao modo como o bebê ocupa seu lugar de enunciação e os sinais relacionados à sustentação enunciativa oferecida pela mãe.

Para a análise estatística foi utilizado o coeficiente de correlação de Spearman (Tabela 1), para verificar as correlações entre os sinais enunciativos e os indicadores do IRDI, considerando as seguintes variáveis:

**Tabela 1:** Coeficiente de correlação de Spearman e significância estatística

Variáveis	Coeficiente de correlação (r)	p-valor*
SEAL bebê x SEAL mãe	0,76	0,000
SEAL bebê x IRDI- SS	0,31	0,006
SEAL bebê x IRDI- ED	0,29	0,011
SEAL bebê x IRDI-PA	0,23	0,048
SEAL bebê x IRDI-FP	0,09	0,460
SEAL bebê x TOTAL IRDI	0,23	0,044
SEAL mãe x IRDI-SS	0,36	0,001
SEAL mãe x IRDI-ED	0,34	0,003
SEAL mãe x IRDI-PA	0,31	0,006
SEAL mãe x IRDI-FP	0,18	0,118
SEAL mãe x TOTAL IRDI	0,31	0,006
TOTAL SEAL x IRDI-SS	0,34	0,002
TOTAL SEAL x IRDI-ED	0,31	0,006
TOTAL SEAL x IRDI-PA	0,26	0,020
TOTAL SEAL x IRDI-FP	0,11	0,338
TOTAL SEAL x TOTAL IRDI	0,27	0,020

* Coeficiente de correlação de Spearman

**Legenda:** SEAL=sinais enunciativos de aquisição da linguagem; IRDI=indicadores clínicos de referência ao desenvolvimento infantil, SS=suposição de sujeito; ED=estabelecimento da demanda, PA=presença/ausência, FP=função paterna. (n=77) p≤0,05

- SEAL do bebê x SEAL da mãe;
- SEAL do Bebê x indicadores do eixo Suposição de Sujeito;
- SEAL do Bebê x indicadores do eixo de Estabelecimento da Demanda;
- SEAL do Bebê x indicadores do eixo de Alternância entre Presença e Ausência;
- SEAL do Bebê x indicadores do eixo Função Paterna;
- SEAL do Bebê x Total do IRDI;
- SEAL da Mãe x indicadores do eixo Suposição de Sujeito;
- SEAL da Mãe x indicadores do eixo de Estabelecimento da Demanda;
- SEAL da Mãe x indicadores do eixo de Alternância entre Presença e Ausência;
- SEAL da Mãe x indicadores do eixo Função Paterna;
- SEAL da Mãe x Total do IRDI;
- SEAL total x indicadores do eixo Suposição de Sujeito;
- SEAL total x indicadores do eixo de Estabelecimento da Demanda;
- SEAL total x indicadores do eixo de Alternância entre Presença e Ausência;
- SEAL total x indicadores do eixo Função Paterna; e
- SEAL total x IRDI total.

Além dessa análise, foram comparados os dados de bebês com (menos de 18 sinais) e sem atraso na aquisição da linguagem (18 ou mais sinais)^(7)^ quanto ao seu desempenho nos eixos do roteiro IRDI (SS, ED, PA, FP) e o total do IRDI por meio do Teste U de Mann-Whitney. Cabe destacar, que foi a partir de um estudo clínico inicial que se observou que crianças com 18 ou mais sinais enunciativos não possuíam atraso na aquisição da linguagem, aos 24 meses. Entretanto, crianças com atraso no processo da aquisição da linguagem tinham 12 sinais enunciativos dos 24 sinais avaliados^(7)^.

## RESULTADOS

Após a análise dos dados dos 77 bebês, observou-se que houve um maior número de bebês que apresentaram risco pelo SEAL (45 bebês), quando comparado ao roteiro IRDI (31 bebês). Na Tabela 1, encontra-se a análise da correlação entre ambos instrumentos.

Pode-se observar, na Tabela 1, que há correlação significativa entre o SEAL do bebê e o SEAL da mãe, confirmando a relação entre o fator materno e o do bebê na geração de atraso na aquisição da linguagem. Essa observação se confirma na correlação entre o SEAL total, o SEAL total do bebê e o SEAL total da mãe.

Em relação aos eixos do roteiro IRDI e o SEAL, observa-se que os eixos suposição de sujeito (SS), estabelecimento de demanda (ED) e alternância entre presença e ausência (PA) se correlacionaram tanto aos sinais maternos, quanto aos do bebê. Não há correlação significativa entre os sinais enunciativos e o eixo função paterna de modo isolado. Também se verificou uma correlação significativa entre Total SEAL e Total IRDI.

Portanto, confirmou-se a correlação entre os indicadores relativos aos eixos de suposição de sujeito, estabelecimento da demanda e alternância entre presença e ausência, que estão relacionados de modo fundamental à função materna.

A Tabela 2 apresenta a comparação das pontuações dos eixos do IRDI entre os grupos do SEAL.

**Tabela 2:** Comparação das Pontuações dos Eixos do IRDI entre os Grupos do SEAL

	Médias		
	SEAL <18	SEAL ≥18	p-valor*
IRDI SS	4,24	4,66	0,021
IRDI ED	9,29	10,19	0,034
IRDI PA	3,11	3,56	0,053
IRDI FP	3,84	4,16	0,631
TOTAL IRDI	14,87	16,31	0,084

* Teste U de Mann-Whitney; p≤0,05

**Legenda:** IRDI=indicadores clínicos de referência ao desenvolvimento infantil, SEAL=sinais enunciativos de aquisição da linguagem. Ponto de corte SEAL= 18 ou mais normal, <18 = risco, SS= suposição de sujeito, ED=estabelecimento da demanda, PA= presença/ausência, FP= Função paterna

Considerando que o ponto de corte para atraso na aquisição da linguagem no SEAL é de 18 sinais^(7)^, observa-se, na Tabela 2, que houve diferença significativa na pontuação dos eixos do roteiro IRDI entre os grupos com e sem atraso na aquisição da linguagem quanto aos eixos de suposição de sujeito (SS), estabelecimento da demanda (ED) e alternância presença/ausência (PA) dos indicadores clínicos de referência ao desenvolvimento infantil.

Além disso, foi possível identificar que não há diferença significativa nas pontuações do eixo função paterna (FP) para casos de o bebê apresentar menos ou mais sinais enunciativos de aquisição da linguagem, bem como do valor total do IRDI nesta análise, o que, de certa forma, vai ao encontro do que foi identificado na análise apresentada na Tabela 1. Assim, pode-se afirmar que a função materna se apresentou especialmente relacionado à ocupação de um lugar de enunciação pelos bebês e/ou sustentação desse lugar por suas mães.

## DISCUSSÃO

Os resultados desta pesquisa, envolvendo SEAL do Bebê, SEAL da Mãe *versus* total SEAL, evidenciaram uma relação dos sinais da mãe e do bebê na geração de atraso na aquisição da linguagem, ou seja, não cabe mais a hipótese de que a geração do atraso na aquisição da linguagem deva-se apenas às condições biológicas do bebê. A sustentação enunciativa do bebê é tão importante quanto as limitações biológicas na geração do atraso na aquisição da linguagem. De modo especial, destaca-se que a alimentação da suposição de falante pelo cuidador^(9)^ é fundamental para que o bebê se engajar em relações conjuntivas iniciais e descobrir os efeitos de suas manifestações no outro por meio de relações disjuntivas^(8)^.

Na correlação e comparação entre casos com atraso e sem atraso na aquisição da linguagem identificados pelo SEAL, observaram-se alterações nos três eixos da função materna SS, ED, PA do roteiro IRDI, tanto nos sinais do bebê, quanto nos da mãe. Isso indica que a suposição de um sujeito, o estabelecimento da demanda do bebê e a alternância entre presença e ausência são fundamentais na criação de um lugar de enunciação para o bebê^(7)^. Os resultados indicam que quando supõe um sujeito, a mãe também consegue supor um falante^(9)^, abrindo espaço para interpretar a partir de gestos e vocalizações do bebê^(10)^, além de suas demandas e guardando lugar para as próximas manifestações desse sujeito, evidenciado na alternância entre ausência e presença^(11)^.

Quanto ao indicador 4 do roteiro IRDI (a mãe propõe algo e aguarda a resposta do bebê), pode-se afirmar que é fundamental na geração do diálogo^(12)^, o que evidencia a importância de a mãe ou seu substituto não ser só presença ou ausência. Esse eixo parece ser necessário para que o bebê possa emergir na relação de disjunção, pois a mãe precisa deixar vago um lugar discursivo ao bebê e ele tem de ocupar esse lugar para emergir na fala.

Já o eixo de função paterna não se apresentou como um gerador isolado do atraso na aquisição da linguagem, mas operou nos outros dois eixos, pois o exercício da função materna está sempre referenciado à função paterna^(13)^. Significa dizer que, ao isolar os eixos do IRDI correspondente à função materna (mãe castrada), a função paterna também opera, embora sua discriminação da função materna seja mais evidente no segundo ano de vida.

Um estudo^(14)^ observou que os bebês com alterações no eixo da função paterna demonstravam uma maior dificuldade em ter uma fala descolada da fala materna, mas não uma dificuldade em falar em si, ou seja, em passar da referência mostrada à falada. A dificuldade maior era em atingir o terceiro mecanismo enunciativo, ou a entrada no próprio discurso, o que não é avaliado no SEAL, já que é uma atividade um pouco mais presente a partir dos 24 meses nos bebês, quando a linguagem é utilizada para imaginar um mundo possível e se observam as instanciações de si próprio no discurso, bem como, o aparelho das funções^(8)^. Talvez, em uma idade acima de dois anos seria possível observar outros efeitos da função paterna na autonomia discursiva, os quais não foram possíveis analisar neste estudo.

Desse modo, a relação entre psiquismo e linguagem demanda que se levante uma hipótese de funcionamento de linguagem, a qual inclua tanto os aspectos psíquicos, quanto os aspectos linguísticos. O modo como os pais sustentam o bebê durante o diálogo sofre efeitos da forma como supõem o filho. Se o filho não é suposto como um sujeito em separado, isso leva a um funcionamento de linguagem em que a mãe pode falar pelo filho, sem lhe dar um lugar de enunciação. Por outro lado, se o filho possui dificuldades em ocupar seu lugar de enunciação, os pais podem falar por ele, em função da ansiedade diante da posição mais silenciosa da criança^(14)^.

Por fim, cabe destacar as limitações deste estudo pelo uso do roteiro IRDI reduzido, embora ele tenha contado com uma boa amostra de caráter longitudinal. Deste modo, recomenda-se a utilização de uma amostra maior para uma análise mais representativa da população. Destaca-se, que essa tendência geral estatística não exclui a possibilidade de casos singulares, em que apenas a função paterna alterada possa gerar um atraso na aquisição da linguagem mais significativo, já detectável no segundo ano de vida.

Ressalta-se ainda, a importância de um acompanhamento tanto pelo fonoaudiólogo, quanto por um psicanalista, desde o primeiro ano de vida da criança, pois, assim, é possível conseguir reconhecer sinais de alerta que são observáveis desde os primeiros meses de vida, pela interação realizada entre o bebê e as figuras parentais. Além disso, para a atuação de detecção precoce de risco ao desenvolvimento infantil é necessário um aporte interdisciplinar, numa perspectiva de promoção de saúde, que vise a qualidade de vida do sujeito, desde seu nascimento^(15)^.

## CONCLUSÃO

Este trabalho buscou analisar a correlação dos eixos teóricos dos Indicadores de Referência ao Desenvolvimento Infantil (IRDI) e as possíveis diferenças nos Sinais Enunciativos de Aquisição da Linguagem (SEAL), quanto ao papel do bebê e da mãe nas protoconversações iniciais. A partir da análise desenvolvida, pode-se afirmar que houve correlação significativa entre os eixos de suposição de sujeito, estabelecimento da demanda e alternância entre presença e ausência do roteiro IRDI e risco à linguagem pelo SEAL. Quando comparados, os bebês com e sem atraso na aquisição da linguagem pelo SEAL, os mesmos eixos se mantiveram significativos na diferenciação dos bebês.
